# Preparation of Hybrid Nanoparticle Nucleating Agents and Their Effects on the Crystallization Behavior of Poly(ethylene terephthalate)

**DOI:** 10.3390/ma11040587

**Published:** 2018-04-11

**Authors:** Zhenzhen Han, Yao Wang, Jiuxing Wang, Shichao Wang, Hongwei Zhuang, Jixian Liu, Linjun Huang, Yanxin Wang, Wei Wang, Laurence A. Belfiore, Jianguo Tang

**Affiliations:** 1 Institute of Hybrid Materials, The National Base of International Scientific and Technological Cooperation on Hybrid Materials, The National Base of Polymer Hybrid Materials in the Programme of Introducing Talents Dicipline to Universities, College of Materials Science and Engineering, Qingdao University, Qingdao 266071, China; 18724713687@163.com (Z.H.); jiuxingwang@foxmail.com (J.W.); wangsc@qdu.edu.cn (S.W.); zhw310310@163.com (H.Z.); ljx@qdu.edu.cn (J.L.); newboy66@126.com (L.H.) Yanxin_2008@126.com (Y.W.); wangwei040901@163.com (W.W.); belfiore@engr.colostate.edu (L.A.B.); 2 Department of Chemical and Biological Engineering, Colorado State University, Fort Collins, CO 80523, USA

**Keywords:** PET, nano-SiO_2_, non-isothermal crystallization, nucleating agent

## Abstract

In this research contribution, the primary objective was to enhance the crystallization behavior of poly(ethylene terephthalate) (PET). To accomplish this tack, three kinds of new nucleating agents SiO_2_-diethylene glycol-LMPET (PET-3), SiO_2_-triethylene glycol–LMPET(PET-4) and SiO_2_-tetraethylene glycol-LMPET (PET-5) nucleating agents were prepared via grafting different oligomers (diethylene glycol; triethylene glycol and tetraethylene glycol) to the surface of nano-SiO_2_ and then linking to the low molecular weight poly(ethylene terephthalate) (LMPET). These nano-particle nucleating agents facilitated the crystallization of PET. Differential scanning calorimetry (DSC) studies of the composites that pure PET blended with PET-3, PET-4 and PET-5 indicated that the longer ethoxy segment in the nucleating agents exhibited (i) higher degrees of crystallinity; (ii) faster rates of crystallization; and (iii) higher crystallization temperatures. The Jeziorny method was employed to analyze the non-isothermal crystallization kinetics of the composites. These works demonstrated that the PET-3, PET-4 and PET-5 were attractive nucleating agents for poly(ethylene terephthalate), and the longer the chain length of the ethoxy segment in the nucleating agents, the more efficient the nucleation effect.

## 1. Introduction

Poly(ethylene terephthalate) (PET) is an important engineering semicrystalline polymer due to its superior thermal and physical properties, excellent chemical resistance, and low permeability, which is widely used in fibers, bottles, and packages, etc. [1,2,3,4,5]. Its low crystallization rate at normal mold temperatures and inherent brittleness characteristics limits the wide application of PET [6,7,8,9]. A variety of inorganic substances are commonly added at small concentrations to produce high crystallinity and rapid crystallization rate [10,11,12,13]. Blending with inorganic nanoparticles is a simple and low-cost method to introduce nucleating agents into PET to increase the mechanical and other properties of polymers [14,15]. Nano-SiO_2_ is widely used as a kind of common additive material in industrial production because of its nontoxicity, environment friendliness, amorphism, small particle size, large specific surface area and large surface activity [16]. PET/Nano-SiO_2_ had been prepared by Antoniadis and Cai, and its crystallization behavior showed that nano-SiO_2_ can act as a nucleating agent [17,18]. However, the nano-SiO_2_ nucleating agent cannot uniformly disperse in the matrix of PET, which can influence the crystallization of PET because the nano-SiO_2_ is of high surface energy and poor compatibility with most materials [19]. Therefore, it needs to be modified. The surface modification of silica mainly involves the chemical reaction and graft polymerization of hydroxyl groups on the surface of silica. The organic molecules which grafted to the silica are generally linear in structure and the organic molecules involved in this work belongs to this class. Such measures can effectively improve the compatibility of the nucleating agents with the polymers and improve the dispersion stability [20,21]. In this research, to enhance the crystallization behavior of PET, a series of different oligomers (diethylene glycol; triethylene glycol; and tetraethylene glycol) were grafted to the surface of nano-SiO_2_ and then linked to the low molecular weight poly(ethylene terephthalate) (LMPET). Finally, SiO_2_-diethylene glycol-LMPET (PET-3), SiO_2_-triethylene glycol-LMPET (PET-4) and SiO_2_-tetraethylene glycol-LMPET (PET-5) were obtained. We introduced the PET-3, PET-4 and PET-5 into PET to improve the dispersion of the nucleating agents in PET and the crystallization properties of PET. LMPET is used to make the nucleating agents more compatible with the PET matrix. As flexible segment, the ethoxy segment is beneficial to the motion of molecular chains during crystallization and thus improves the crystallization properties. Furthermore, the effects of the nucleating agents on the non-isothermal kinetics of the composites were studied by the Jeziorny method.

## 2. Materials and Methods

### 2.1. Materials

The nano-SiO_2_ was purchased from Zhejiang Yuda Chemical Co. Ltd. (Shaoxing, China) and its particle size is less than 30nm with a specific surface area of 200 m^2^·g^−1^. It should be dried under vacuum at 60 °C before use. Diethylene glycol (DEG) and thionyl chloride (TEG) was purchased from Tianjin Bodi Chemicals Co. Ltd. (Tianjin, China). Zincacetate were purchased from Tianjin Guang Cheng Chemicals Co. Ltd. (Tianjin, China). Benzene and ethylene glycol were purchased from Tianjin Fuyu Fine Chemical Co. Ltd. (Tianjin, China). Phenol was purchased from Tianjin Yongda Chemical Reagent Co. Ltd. (Tianjin, China). Tetrachloroethane was purchased from Shanghai Sanpu Chemical Co. Ltd. (Shanghai, China). Triethylene glycol and antimony trioxide was purchased from Tianjin Daochem Chemical Reagent Co. Ltd. (Tianjin, China). Tetraethylene glycol was purchased from Macklin Biochemical Co., Ltd. (Shanghai, China). Toluene was purchased from Yantai Shuangshen Chemical Co. Ltd. (Yantai, China). Dimethyl terephthalate (DMT) was purchased from Sinopharm Group Chemical Reagent Co. Ltd. (Suzhou, China).

### 2.2. Synthesis

#### 2.2.1. Preparation of Silicon Chloride

Under the protection of nitrogen gas, 2.5 g of nano-SiO_2_ was dispersed into 25 mL of benzene solvent and then 25 mL of thionyl chloride was added to the flask to react for 4 h at 65 °C. Next, the mixture was centrifuged at a speed of 8000 r·min^−1^ for 15 min and washed by benzene to remove the non-reactive thionylchloride. Finally, the product of silicon chloride was dried under vacuum at 80 °C for 48 h.

#### 2.2.2. Preparation of SiO_2_-diethylene glycol/SiO_2_-triethylene glycol/SiO_2_-tetraethylene glycol

Under the nitrogen atmosphere, 2 g of silicon chloride was dispersed into 25 mL toluene and 11.94 g of diethylene glycol/16.89 g of triethylene glycol/21.85 g of tetraethylene glycol was added into the solution and reacted for 5 h at 65 °C with magnetic stirring. After the reaction completed, the mixture was centrifuged at a speed of 8000 r·min^−1^ and washed with toluene and acetone successively. Finally, the product was dried in vacuum at 80 °C for 48 h to obtain dried SiO_2_-diethylene glycol/SiO_2_-triethylene glycol/SiO_2_-tetraethylene glycol samples. 

#### 2.2.3. Preparation of SiO_2_-diethylene glycol-LMPET/SiO_2_-triethylene glycol-LMPET/SiO_2_-tetraethylene glycol-LMPET 

**Figure 1:**
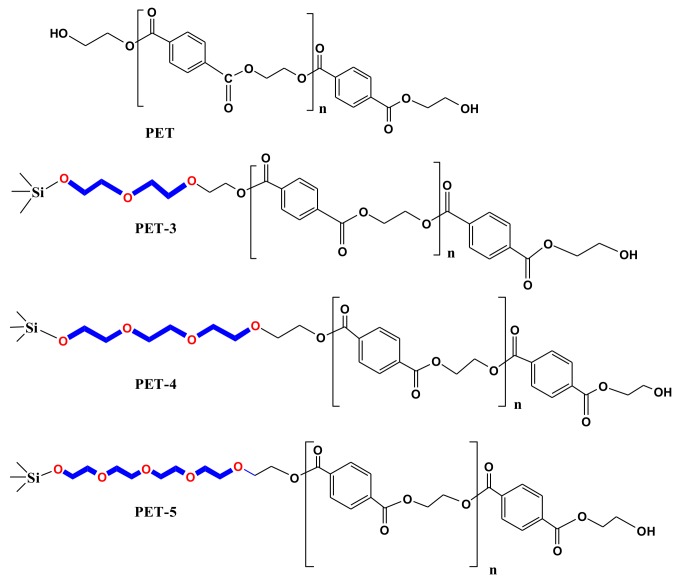
Chemical structures of PET, PET-3, PET-4 and PET-5.

10 g of LMPET was dissolved in a mixed solvent of phenol and tetrachloroethane with the mass ratio of 1:1 (40 g respectively) and then 5 mL of cross-linking agent ethylene glycol was added dropwise. The reaction was carried out at 60 °C for 2 h. Subsequently, 0.03 g of polycondensation catalyst Sb_2_O_3_ and 1 g of SiO_2_-diethylene glycol/SiO_2_-triethylene glycol/SiO_2_-tetraethylene glycol samples were added into the mixture to react for 2 h at 100 °C. After the reaction completed, the mixture was centrifuged at the speed of 8000 r·min^−1^ and washed in a mixed solvent of phenol and tetrachloroethane with the mass ratio of 1:1. Then, the product was dried in vacuum at 80 °C and we got SiO_2_-diethylene glycol-LMPET/SiO_2_-triethylene glycol-LMPET/SiO_2_-tetraethylene glycol-LMPET (abbreviated as PET-3, PET-4 and PET-5 in Figure 1).

#### 2.2.4. Preparation of PET/PET-3, PET/PET-4, PET/PET-5

**Figure 2:**
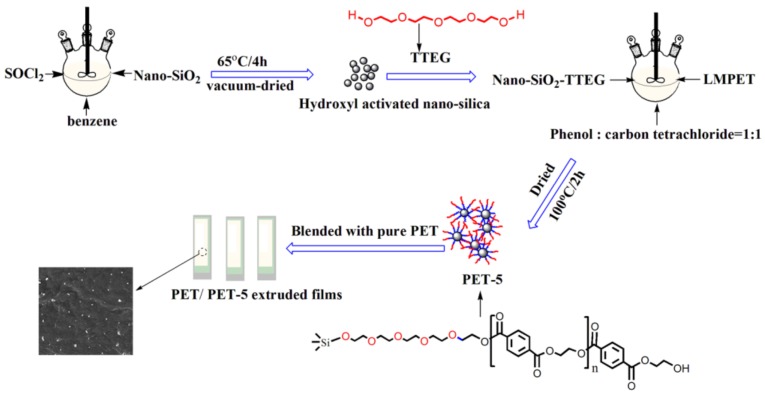
Scheme of interaction mechanism between PET and PET-5.

2 wt % (2 g) of PET-3, PET-4 and PET-5 were mixed with PET pellets (98 g) in twin screw extruder at 275 °C to produce PET/PET-3, PET/PET-4 and PET/PET-5 polymer composite chips, respectively. Figure 2 shows the scheme of interaction mechanism between PET and PET-5.

### 2.3. Characterizations

^1^H-NMR spectra of the nucleating agents were recorded on a Bruker AVANCE-400 NMR nuclear magnetic resonance instrument (Shimadzu, Kyoto, Japan) which used chloroform as the solvent and tetramethylsilane (TMS) as the internal reference at room temperature. DSC studies were carried out using DSC 821e, (Mettler-Toledo, Zurich, Switzerland) under nitrogen atmosphere and the sample mass was kept at 8–10 mg. Each sample was heated to 300 °C and keep insulated for 5 min to eliminate the thermal history. Then the samples were heated and cooled at constant rates of 5/10/15/20 °C·min^−1^, respectively. Field emission scanning electron microscopy was carried on FESEM, JSM-7500F microscope (JEOL, Akishima-shi, Japan). Thermogravimetric analysis (TGA) was performed on a Perkin-ElmerDiamond TGA calorimeter (Perkin-Elmer, Waltham, MA, USA) under a nitrogen purge stream at a flow rate of 20 mL·min^−1^. Vacuum dried samples were subjected to be heated from 25 °C to 700 °C at the rate of 10 °C·min^−1^. X-ray diffraction analysis was performed at room temperature by using an XRD D-8, (Brook, Karlsruhe, Germany). The experiments were operated from 2θ = 0° to 80° at a scanning rate of 2°·min^−1^.

## 3. Results and Discussion

**Figure 3:**
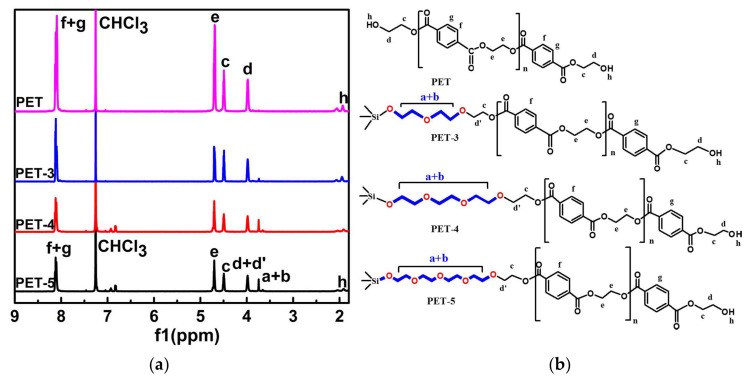
The ^1^H-NMR spectra of PET, PET-3, PET-4 and PET-5 (**a**); structural formula with corresponding proton (**b**).

^1^H-NMR spectra for PET, PET-3, PET-4 and PET-5 are illustrated in Figure 3a and the corresponding assignments of the peaks are marked in the structural formula (Figure 3b). The chemical shift at δ = 8.1 ppm is the characteristic absorption bands on the benzene ring (f, g). The chemical shift at δ = 7.26 ppm is related to the proton of CHCl_3_. The chemical shift at δ = 4.5 ppm is corresponded to the proton (c) in LMPET. The OCH_2_ resonances for LMPET are observed at 4.0–4.7 ppm (c, d, and e) due to the influence of ester groups. The weak OCH_2_ resonance (d’) represents the link between LMPET and the ethoxy segment. The ^1^H resonance at 3.63 ppm is assigned to proton (a, b) in the ethoxy segment which links the SiO_2_ with LMPET, obviously, the peak of a + b strengthened with the increased length of the ethoxy segment in the nucleating agents.

**Figure 4:**
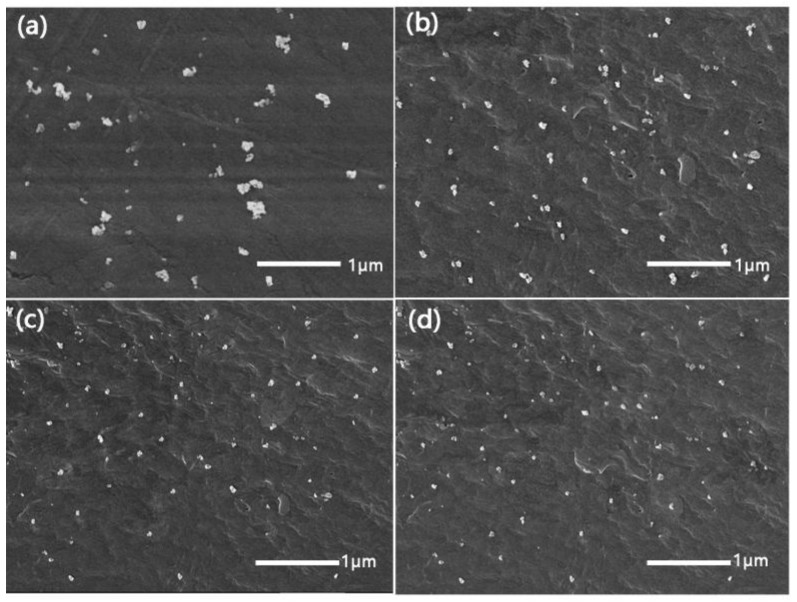
FESEM images of composites (**a**) PET/SiO_2_; (**b**) PET/PET-3; (**c**) PET/PET-4 and (**d**) PET/PET-5.

From the high-magnification field emission scanning electron microscopy (FESEM) images, as shown in Figure 4, we can see that the bare nano-SiO_2_ were agglomerated in PET which was similar to Antoniadis’ study on the non-isothermal crystallization kinetic of PET/SiO_2_ [17]. The agglomeration of PET-3, PET-4 and PET-5 dispersed in PET was reduced and the partial size of nucleating agents was 50–70 nm, due to the grafting of diethylene glycol-LMPET, triethylene glycol-LMPET and tetrathylene glycol-LMPET outside the nucleating agents which made the nucleating agents more compatible with the PET matrix.

**Figure 5:**
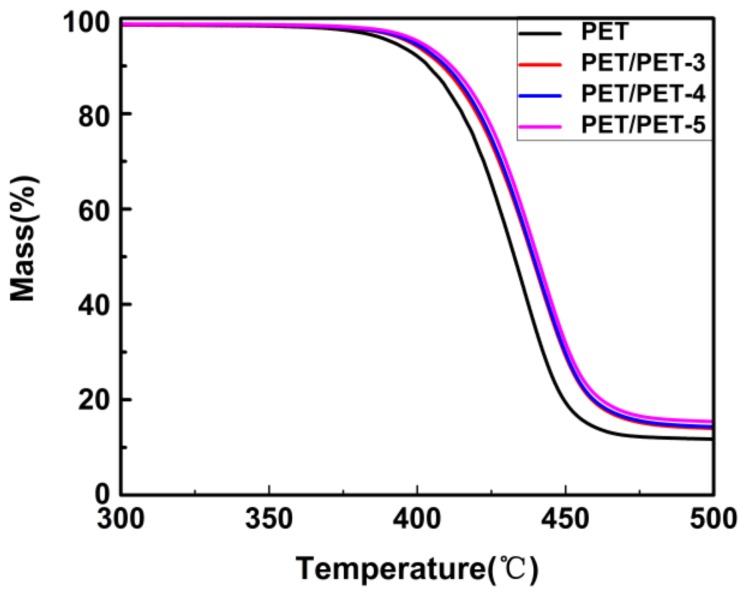
TGA curves of pure PET, PET/PET-3, PET/PET-4 and PET/PET-5.

The thermal decomposition curves of PET and its composites are shown in Figure 5. As we can see, the addition of the nucleating agents decreased the final weight loss of PET from 88% to 84%. Meanwhile, the initial decomposition temperature of the composites increased from 375 °C to 393 °C, which implied that the addition of the nucleating agents improved the thermal stability of PET.

**Figure 6:**
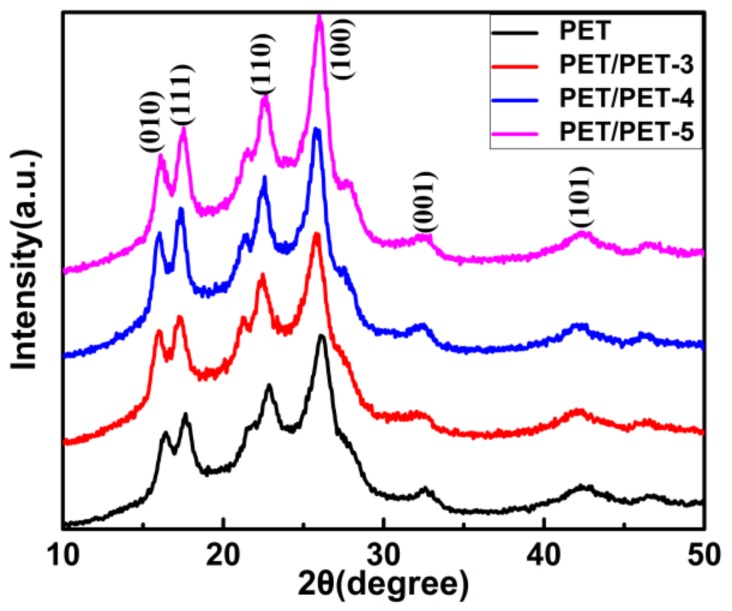
The XRD patterns of PET, PET/PET-3, PET/PET-4 and PET/PET-5.

X-ray powder diffraction (XRD) was applied to study the influence of PET-3, PET-4 and PET-5 on the crystalline structure of PET. Figure 6 shows the XRD patterns of pure PET and its composites. At 2θ = 16.3°, 17.6°, 22.7°, 25.9°, 32.6° and 42.2°, the diffraction peaks corresponded to (010), (111), (110), (100), (001) and (101) planes of PET, respectively, which also occurred at the same 2θ of the composites and suggested that the nucleating agents did not change the crystalline structure of PET [22]. The small peak at 2θ = 21.5° was caused by the (111) plane of SiO_2_ while the small peak occurred at the right of the (100) was related to the ethoxy segment grafted on the surface of SiO_2_.

**Figure 7:**
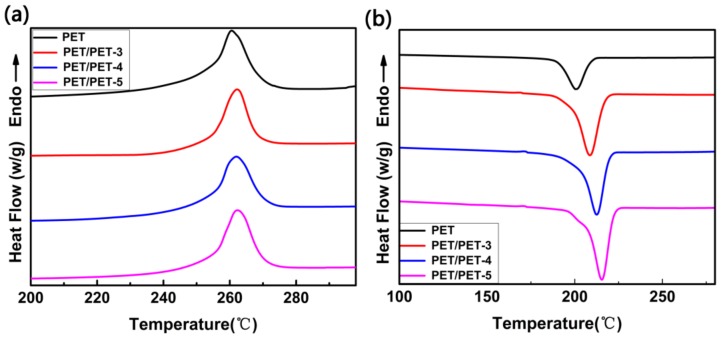
The heating curve (**a**) and the cooling curve (**b**) at the rate of 10 °C·min^–1^.

Figure 7b shows the DSC curves of PET composites with different nucleating agents at the cooling rate of 10 °C·min^−1^. With the increased chain length of the ethoxy segment in the nucleating agents, the crystallization peak temperature (T_c_) of the composites increased gradually and the PET/PET-5 showed the most increase, approximately 16.9 °C, implying that PET-3, PET-4 and PET-5 can act as nucleating agents to accelerate the crystallization of PET and PET-5 is the most effective. Because with the increased chain length of ethoxy segment grafted on the surface of SiO_2_, the interaction between SiO_2_ and PET molecular chains increased a lot, which is benefit for the winding of PET molecular chains on the surface of the nucleating agent. On the other hand, as the flexible segment, the ethoxy segment is beneficial to the motion of the nucleating agents and it is easier for the PET molecules to attach to the surface of the nucleating agents and form nuclei. Furthermore, the formed nuclei induce the crystallization of PET. In Figure 7a, the melting point of the samples were improved gradually with the addition of the nucleating agents because the nucleating agents made the crystal structure of PET more perfect.

The degree of crystalline (X_c_) at the test rate of 10 °C·min^–1^ was also calculated by Equation (1) [23]:

(1) $$X_{c}=\frac{\Delta H_{f}}{\left(1-\omega \right)\Delta H_{f}^{0}}\times 100\text{\%}$$

Where ΔH_f_ is the measured heat of melting and $\Delta H_{f}^{0}$ is the heat of fusion of 100% crystallinity (140 J·g^−1^) [22]. Obviously, the crystallinity of composites increased with the addition of nucleating agents (listed in Table 1), and corresponding to this result, both of the ∆H_m_ and the ∆H_c_ increased. With the increased chain length of ethoxy segment in the three kinds of nucleating agents, the nucleating effect was enhanced. The 2 wt % of PET-5 increased the crystalline of PET by 7%.

The Jeziorny method (Equations (2)–(4)) was used to analyze the isothermal crystallization [24]:

(2) $$1-X_{t}=\exp\left(-{Z\;t}^{n}\right)$$

(3) ln[−ln(1−X(t))]=nlnt+lnZ

(4) $${\mathrm{lnZ}}_{c}=\left(\mathrm{lnZ}\right)/\beta$$

where n is the Avrami index related to the nucleation mechanism and the growth mode of the crystal. Z_c_ is the crystallization rate constant and X(t) is the relative crystallinity at different times calculated according to the Formula (5):

(5) $$X_{t}=\int_{t_{0}}^{t}\left(\frac{{\mathrm{dH}}_{c}}{\mathrm{dt}}\right)\mathrm{dt}/\int_{t_{0}}^{\infty }\left(\frac{{\mathrm{dH}}_{c}}{\mathrm{dt}}\right)\mathrm{dt}$$

Where dH/dt is the heat flow rate; and T_0_ and $T_{\infty }$ are the time at which crystallization starts and ends, respectively.

**Figure 8:**
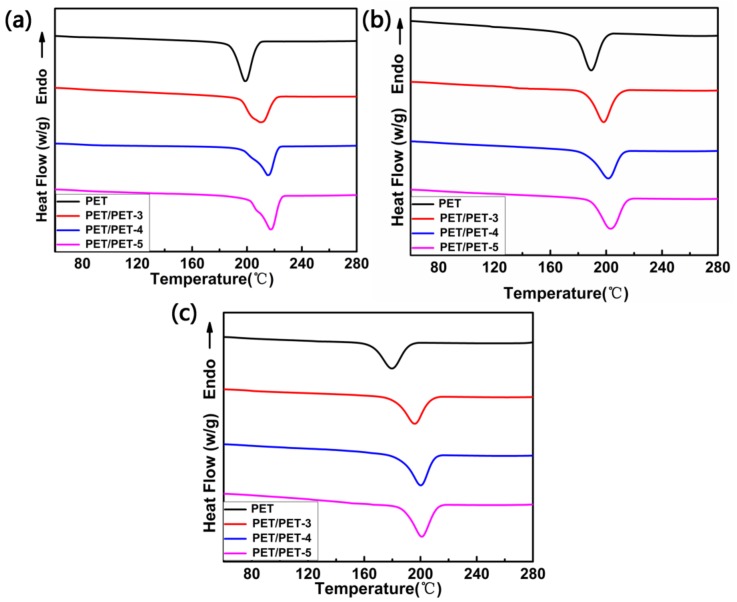
DSC curves for PET, PET/PET-3, PET/PET-4 and PET/PET-5 at different cooling rates. (**a**) 5 °C·min^−1^; (**b**) 15 °C·min^−1^ and (**c**) 20 °C·min^−1^.

To analyze the isothermal crystallizations of PET and its composites in the Jeziorny method, DSC curves for all the samples at different cooling rates were tested in Figure 7b and Figure 8. At the same cooling rate, the crystallization peak temperature (T_c_) shift to higher values gradually with the addition of the nucleating agents PET-3, PET-4 and PET-5.

**Figure 9:**
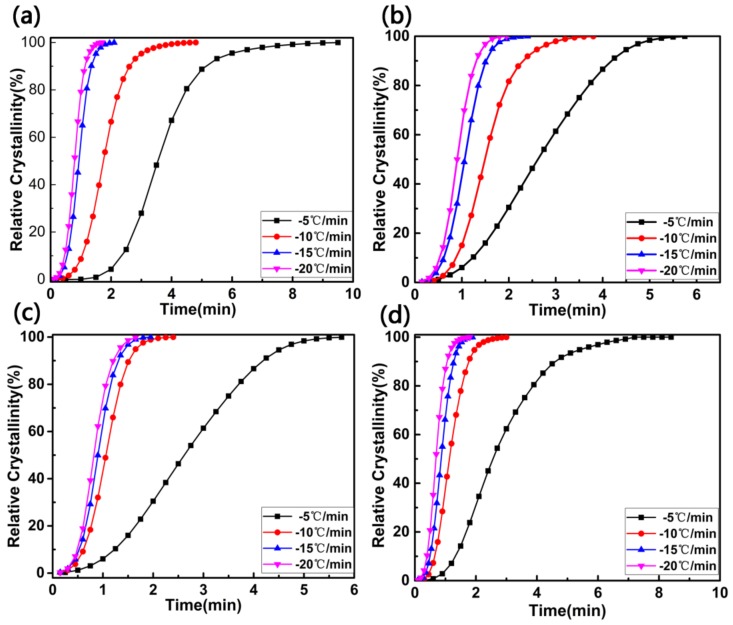
Plots of relative degree of crystallinity (X_t_) versus crystallization time (t) for (**a**) PET; (**b**) PET/PET-3; (**c**) PET/PET-4 and (**d**) PET/PET-5 at different cooling rates.

Based on the DSC crystallization curves, non-isothermal crystallization process at different cooling rates shown in Figure 9 were obtained according to Formula (5) and an important parameter that can be taken directly is the value of t_1/2_ (the time at which the relative crystallinity of the polymers achieves 50% of the total crystallinity measured at that temperature and can reflect the overall crystallization rate of the polymers). Generally, the t_1/2_ is directly used to characterize the crystallization rate, and the low t_1/2_ values predict high crystallization rate. Obviously, at the same cooling rate, the t_1/2_ values decreased in the order of PET/PET-3, PET/PET-4 and PET/PET-5, suggested a higher crystallization rate was caused with the increased length of ethoxy segment in the nucleating agents.

**Figure 10:**
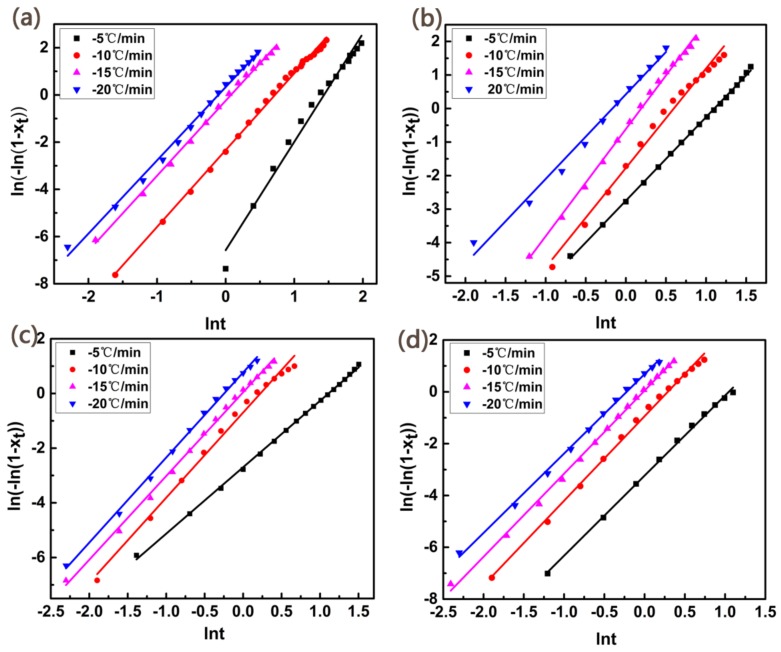
Plots of ln[−ln(1 − X_t_)] vs. lnt for (**a**) PET; (**b**) PET/PET-3; (**c**) PET/PET-4 and (**d**) PET/PET-5 at different cooling rates.

The plots of ln[−ln[(1 − X_t_)] vs. lnt are shown in Figure 10. At the early crystallization process, the plots were presented favorable linear relationship, suggesting that the Jeziorny method was relatively appropriate in describing the non-isothermal crystallization kinetics. The value of n and Z_c_ (determined from the slope and intercept respectively) are listed in Table 2. The Avrami index, to a certain extent, can reflect the nucleation mechanism and the growth mode of crystallization. The value of n is composed by the space dimension of growth and the time dimension of nucleation. Homogeneous nucleation has dependence on the time and the time dimension is 1. However, for heterogenous nucleation, there is no dependence on the time dimension, so the time dimension is 0. Normally, n = 4 represents the homogeneous nucleation with three-dimensional growth while n = 3 represents heterogeneous nucleation with three-dimensional growth. The values of n for composites were not integer because the initial nucleation for the crystallization of PET is dependent on the time, the simultaneous occurrence of homogeneous nucleation and heterogenous nucleation, the simultaneous growth of two-dimensional and three-dimensional spherules, linear growth rate of crystal fluctuates unstably and the secondary crystallization. For PET, the values of n were 3.1–3.9, which indicates that the nucleation of pure PET may be homogeneous with three-dimensional growth. While the values of n for the composites were 2.9–3.3, implying that the crystal growth of composites was likely linear growth (two-dimensional and three-dimensional growth) after heterogeneous nucleation [25,26,27,28,29,30]. The values of Z_c_ increased with the addition of nucleating agents at the same cooling rate, suggesting a higher crystallization rate was caused compared with PET.

## 4. Conclusions

Three hybrid nanoparticle nucleating agents were synthesized and blended with pure PET. Calorimetry studies demonstrated that the crystallinity, crystallization rate and crystallization peak temperature of composites shifted to higher values with the increased chain length of the ethoxy segment in nucleating agents compared with pure PET. Thereby, PET-5 enabled the maximum increment for T_c_ from 199 °C to 216 °C, the X_c_ from 23% to 30% at the cooling rate of 10 °C·min^−1^. Moreover, the XRD result suggested that these nano-particle nucleating agents produce no changes in Bragg diffraction peaks. The Jeziorny method revealed the nucleation mechanism of the samples and confirmed that all three nucleating agents accelerated the crystallization rate of PET. In summary, PET-3, PET-4 and PET-5 can serve as nucleating agents to promote the crystallization of PET and the longer-chain length of the ethoxy segment in the nucleating agents indicated the more effective the nucleation was. This regularity provided a direction for future research on the preparation of more effective PET nucleating agents.

## Figures and Tables

**Table 1:** Thermo-performance parameters of the samples.

Sample	T_c_ (°C)	$\Delta H_{f}$ (J·g^−1^)	∆H_c_ (J·g^−1^)	X_c_ (%)
PET	199.2	32.1	40.1	23
PET/PET-3	208.8	37.1	42.3	27
PET/PET-4	212.5	38.9	43.8	29
PET/PET-5	216.1	40.9	45.5	30

**Table 2:** Non-isothermal crystallization kinetic parameters based on the Jeziorny method.

Samples	β (°C/min)	n	Z_c_
PET	−5	3.9	0.24
	−10	3.3	0.79
	−15	3.2	0.87
	−20	3.1	1.01
PET/PET-3	−5	2.9	0.52
	−10	3.2	0.83
	−15	3.1	0.98
	−20	2.9	1.10
PET/PET-4	−5	2.9	0.57
	−10	3.0	0.90
	−15	3.1	1.04
	−20	3.1	1.13
PET/PET-5	−5	3.1	0.68
	−10	3.0	0.97
	−15	3.3	1.08
	−20	3.2	1.23
